# Frequency of Owner-Reported Bacterial Infections in Pet Guinea Pigs

**DOI:** 10.3390/ani9090649

**Published:** 2019-09-04

**Authors:** Shannon Roberts-Steel, James A. Oxley, Aisling Carroll, Alison P. Wills

**Affiliations:** 1 Animal Welfare Research Arena, Department of Animal and Agriculture, Hartpury University, Hartpury, Gloucestershire GL19 3BE, UK (S.R.-S.) (A.C.); 2 Independent Researcher, Measham, Swadlincote DE12 7LQ, UK

**Keywords:** bacterial infections, guinea pigs, husbandry, welfare

## Abstract

**Simple Summary:**

Guinea pigs are common pets but are susceptible to a range of diseases, some of which can be rapidly fatal without prompt veterinary attention. Bacterial infections are one cause of respiratory and gastrointestinal disease in guinea pigs, but few studies have reported how commonly these infections occur in pet guinea pigs or which types of bacterial infection are most often seen. A survey of guinea pig owners was conducted to assess the types of infections and signs of illness pet guinea pigs had suffered from. From the 524 responses it was determined that just under 40% of owners reported that their guinea pig had been diagnosed with a bacterial infection by their veterinarian. Upper respiratory tract infections were most common followed by urinary tract infections and gastrointestinal infections. Guinea pigs may not tolerate some antimicrobial drugs well due to disruption of the bacteria within their gut, therefore treatment of these types of infection can be challenging even if diagnosed early. Based on the frequency of bacterial infections reported by owners in this study, it is suggested that guinea pig owners practice good hygiene and husbandry and remain vigilant for signs of ill health in their pets.

**Abstract:**

Domestic guinea pigs suffer morbidity and mortality due to a range of bacterial infections amongst other causes. Microorganisms such as *Bordetella bronchiseptica* and *Streptococcus pneumoniae* are commonly implicated in respiratory disease; however, there is a lack of research surrounding the prevalence of these bacterial infections. The aim of this study was to investigate the frequency with which owners reported bacterial infections in pet guinea pigs and to assess owner knowledge of correct husbandry practices to inform prevention of the development of bacterial infections. An online questionnaire, consisting of 30 questions was promoted to guinea pig owners. Of all respondents (*n* = 524), 39.39% reported that their guinea pig(s) had been clinically diagnosed with a bacterial infection, with upper respiratory tract infections the most commonly reported (46.95%), followed by urinary tract (15.49%) and then gastrointestinal infections (11.73%). Owners demonstrated knowledge of correct husbandry practices and there was no significant effect (*p* = 0.475) of owner knowledge on having owned a guinea pig with a bacterial infection. Bacterial infections may be more common in guinea pigs than was previously thought. Further research is required to understand why bacterial infections are common in pet guinea pigs and to help owners to prevent and recognize these infections.

## 1. Introduction

Guinea pigs are frequently kept as household pets [1], yet they are susceptible to a range of health problems. Bacterial infections have been identified within laboratories, breeding colonies and domestic pets and are sometimes associated with poor husbandry and hygiene [2]. Recent prevalence data for bacterial infections in domestic pet guinea pigs is limited, yet these infections can result in significant morbidity in addition to sudden mortality in some cases.

In a retrospective study of 1000 guinea pigs, odontogenic, retrobulbar and skin abscesses were identified in 3.1%, 2.1% and 2.0% of guinea pigs respectively [3]. Gastrointestinal disease was identified in 13.1% of guinea pigs, with 84 guinea pigs displaying enteropathy in association with enteritis [3]. Only 4.0% of the population had been diagnosed with pneumonia, and it was concluded that damp housing conditions can be a predisposing factor [3]. Key bacterial pathogens that have been associated with upper respiratory tract infections (URTIs) and pneumonia in guinea pigs include *Streptococcus equi* subs *zooepidemicus*, *Streptococcus pneumoniae* and *Bordetella bronchiseptica* [4]. *Salmonella* infections have been reported in research colonies, and *Staphlyococcus aureus* can result in acute dermatitis of the forepaws in animals kept in inappropriate housing or environments with poor husbandry [5]. Chlamydial conjunctivitis can also occur in guinea pigs, particularly alongside respiratory tract disease [4].

Health monitoring in guinea pigs is challenging as they frequently do not show signs of illness in order to reduce their susceptibility to predation [6]. Common presentations seen in sick guinea pigs suffering from bacterial infections are reduced physical activity and social interaction progressing to lethargy and anorexia [7]. Reduced physical activity can induce gut stasis which can cause rapid deterioration resulting in sudden death [8].

Changes in housing, social interaction, bedding, hygiene and nutrition are important factors linked to the induction of stress and transmission of bacterial infections in guinea pigs [9]. The optimal environmental temperature range for guinea pigs is 18–26 °C, and they should not be exposed to extremes of either temperature or humidity [10,11]. Appropriate husbandry practices, for example, the use of disinfectants in housing environments are recommended to decrease or eliminate potential pathogens [12].

Guinea pigs can be challenging patients as latent infections and complex differential diagnoses make the treatment of bacterial infections difficult [13]. As guinea pigs can deteriorate quickly when ill, treatment of bacterial illnesses has to be instigated as soon as possible in order to improve the prognosis for the patient [11]. This may result in broad-spectrum antibiotics being prescribed, these tend to be fluoroquinolones, trimethoprim-sulpha and chloramphenicol [4,14]. Lack of knowledge of the underlying pathogen may render these interventions ineffective, in addition to dysbiosis leading to complications such as anorexia and diarrhoea [15]. Guinea pigs are sensitive to fatal dysbiosis associated with antibiotic use of penicillin, macrolides and lincosamides [16,17,18]. Accurately identifying a causative microorganism is time consuming and lacks feasibility when a patient is experiencing an acute infection [19].

The aim of this study was to investigate the frequency of owner reported common bacterial infections in domestic guinea pigs and to investigate owner knowledge of health, hygiene and husbandry factors related to the prevention of bacterial infections.

## 2. Materials and Methods

Research was conducted in line with institutional ethical guidelines (code: ETHICS2018-65). Inclusion criteria required respondents to currently own or have previously owned guinea pigs, and to be over the age of eighteen. Personal information was kept to a minimum, anonymised and stored on a password-protected computer. Participants could withdraw their responses up to a designated cut-off point and all respondents read a paragraph at the start of the questionnaire that stated that by continuing to complete the survey they were providing their informed consent.

### 2.1. Questionnaire Design

A thirty question survey that consisted of closed and open ended questions was devised based on previous studies investigating owner knowledge of pet husbandry and welfare [20,21]. Questions included Likert scales, multiple choice and options for owners to provide qualitative comments. The questionnaire was created on SurveyMonkey™ (www.surveymonkey.com) and advertised online via social media and guinea pig forums allowing a wide range of individuals to be recruited through convenience sampling. The questionnaire was online for a period of three months from October 2016 to January 2017.

### 2.2. Owner Demographics

Owners were presented with five multiple choice questions asking them to state their sex, age, the length of time for which they had cared for guinea pigs, the number of children living in their household (if any) and where they obtained their guinea pig(s).

### 2.3. Clinical History of Guinea Pigs

Participants were asked to provide information on the clinical history of their guinea pig(s). It was explicitly asked whether their guinea pigs had received a diagnosis of a bacterial infection from their veterinarian, if this was the case, then respondents were asked a further question about the type of infection (respiratory, enteritis etc.). If participants selected “other” for the type of bacterial infection their guinea pig had suffered from, they were able to enter details into an open-ended text box.

### 2.4. Owner Knowledge

Participants were asked to rate their level of agreement on a five-point Likert scale (strongly disagree to strongly agree) with fourteen different statements pertaining to guinea pig health and husbandry (some correct, some incorrect in the context of disease transmission; Table 1). In addition, nine questions assessing knowledge of transmission, clinical signs and preventative measures for common bacterial pathogens (*Streptococcus pneumoniae*, *Bordetella bronchiseptica* and *Salmonella*) were posed with multiple choice style answers. For multiple-choice questions, there was only a single correct answer. Respondents were able to skip any question. A knowledge score was calculated for each participant based on their answers to all twenty-three knowledge based questions [20,22]. This score incorporated responses to both the Likert scale and multiple-choice questions. For Likert scale questions, respondents were given two points for strongly agreeing with a correct statement, one point for agreeing, no points for neither agreeing nor disagreeing, minus one point for disagreeing and minus two points for strongly disagreeing. For incorrect statements, answers were reverse coded (e.g., plus two points for strongly disagreeing with an incorrect statement). For the multiple-choice questions, respondents were given one point for identifying the correct answer but there was no negative credit awarded for getting a wrong answer. Respondents gained no credit if they skipped a question. The maximum achievable score was thirty-seven and the minimum score possible was minus twenty-eight.

### 2.5. Data Analysis

All statistical analyses were performed using R version 3.4.2 (packages: plyr, mass) [23]. Chi-square tests were used to test for a significant effect of demographic factors on whether respondents owned a guinea pig that had been clinically diagnosed with a bacterial infection. A Mann–Whitney U test was used to test for a difference in knowledge score between those respondents that had and had not owned a guinea pig that had been clinically diagnosed with a bacterial infection. Kruskal–Wallis and Mann–Whitney U tests were used to test for an effect of demographic factors on knowledge score. The criterion of significance applied was *p* < 0.05.

## 3. Results

### 3.1. Demographic Information

In total, 524 online surveys were completed by owners; 93.70% (491) of respondents were female. The majority (27.17%; 141) of participants were aged between 20 and 30 years old, with 24.28% (126) aged between 41 and 50, 23.89% (124) aged between 31 and 40, 10.98% (57) aged between 51 and 60, with 9.83% (51) under the age of 20 and 3.85% (20) over the age of 60. The majority of respondents (66.79%; 350) did not have any people under the age of 18 in their household; however, 14.89% (78) did have one juvenile living with them. The majority of owners (51.53%; 270) reported that they had cared for guinea pigs for over four years. Guinea pigs were most frequently obtained from a pet shop (41.79%; 219) followed by adoption centre (34.73%; 182). Other methods of obtaining animals included from breeders (12.98%; 68) and friends (9.73%; 51).

There was no significant effect of sex (X^2^_1_ = 0.002, *n* = 523, *p* = 0.966) or owner age (X^2^_5_ = 1.974, *n* = 524, *p* = 0.853) on whether respondents had owned a guinea pig that had been clinically diagnosed with a bacterial infection There was no significant effect of length of time of guinea pig ownership (X^2^_4_ = 3.091, *n* = 524, *p* = 0.543) or the number of children in the household (X^2^_4_ = 0.631, *n* = 524, *p* = 0.960) on whether respondents had owned a guinea pig that had been clinically diagnosed with a bacterial infection. There was no significant effect of where guinea pigs had been obtained on whether respondents had owned a guinea pig that had been clinically diagnosed with a bacterial infection (X^2^_3_ = 3.612, *n* = 524, *p* = 0.307).

### 3.2. Owner-Reported Bacterial Infections

**Figure 1:**
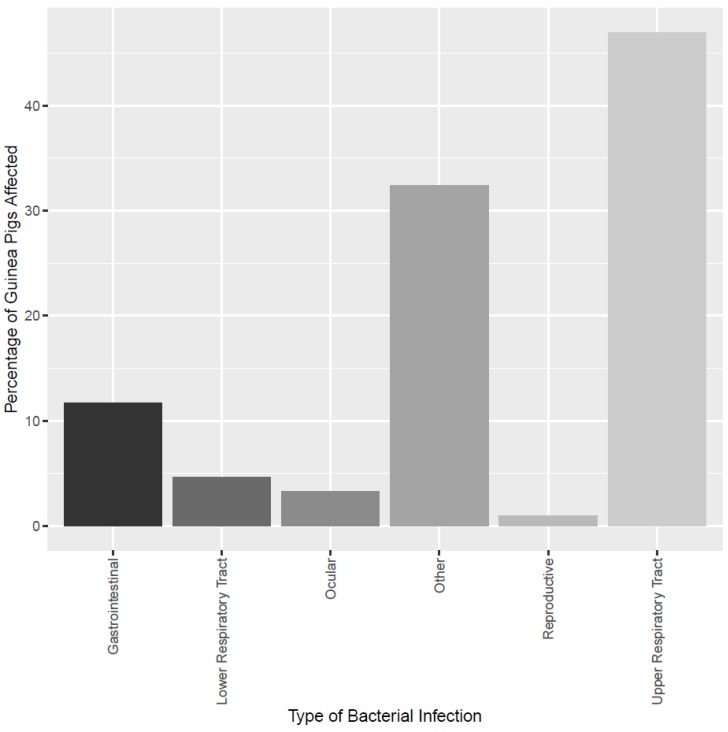
Different types of bacterial infection present in the sample population. Types of bacterial infection and the percentage (out of a total of *n* = 213) for guinea pigs that were reported as having been clinically diagnosed with a bacterial infection.

In this survey, 39.31% (206) of respondents reported having a guinea pig that had been diagnosed with a bacterial infection by their veterinarian. Whilst 206 respondents stated that their guinea pig had received a clinical diagnosis, 213 respondents selected a specific type of bacterial infection in the subsequent question. This may reflect owners not having received a clinical diagnosis but still believing their guinea pig to have suffered from a bacterial infection. Of the seven respondents who answered “no” to having received a clinical diagnosis, six chose the ”other” category when selecting a type of infection and one selected “URTI”. The most frequently reported bacterial infections were URTIs (46.95%; 100); followed by “other” (32.39%; 69), then gastrointestinal (11.73%; 25), lower respiratory tract (4.69%; 10), ocular (3.29%, 7) and reproductive infections (0.94%, 2) (Figure 1). Guinea pigs diagnosed with urinary tract infections (UTI) represented 47.83% (33) of the “other” category, and 15.49% (33) of all infections reported. Other reported infections included dermatological infections, post-operative infections, abscesses and ear infections. The majority of owners (53.63%; 281) strongly agreed that bacterial infections were a concern for guinea pig health.

### 3.3. Owner Knowledge of Housing Factors Relating to the Development of Bacterial Infections

The majority of respondents (55.19%; 282) strongly agreed that guinea pigs should live in a well-ventilated area, with 35.03% (179) agreeing and only 4.89% (25) disagreeing or strongly disagreeing with the statement. The majority of respondents strongly disagreed that wire mesh was an appropriate flooring for a hutch or run (80.59%; 411), with only 2.16% (11) of owners agreeing or strongly agreeing with the statement. The majority of owners strongly disagreed that it was acceptable for guinea pigs to be kept with rabbits (61.33%; 314), with 23.44% (120) disagreeing with the statement and only 6.64% (34) of respondents agreeing or strongly agreeing that rabbits and guinea pigs could be kept together. The majority of owners strongly disagreed that is was acceptable for guinea pigs to be exposed to other rodent species (45.81%; 235), with 35.87% (184) disagreeing that it is acceptable.

The majority of owners (77.95%; 399) agreed that guinea pigs should be housed with another guinea pig provided dominance is not expressed. The majority of owners (36.59%; 187) disagreed that moving guinea pigs between an indoor and outdoor environment on a weekly basis would not cause them stress, 31.31% (160) neither agreed nor disagreed and 21.53% (110) strongly agreed that frequent changes in environment would not result in stress. A high proportion of owners strongly agreed or agreed that extra insulation was not required for guinea pigs that live outdoors (94.53%; 484).

### 3.4. Owner Knowledge of Dietary Factors Relating to the Development of Bacterial Infections

The majority of owners (40.00%; 204) strongly disagreed that washing fruit and vegetables prior to serving was not important. Only 15.29% (78) respondents agreed or strongly agreed that washing fruit and vegetables was not important. In addition, the majority of owners neither agreed nor disagreed (33.79%; 173) that it is not necessary to serve fruit and vegetables at room temperature, with 26.76% (137) agreeing, and 2.73% (14) strongly agreeing that vegetables do not need to be brought to room temperature. Of the owners, 28.13% (144) disagreed that vegetables do not need to be served at room temperature and 8.59% (44) strongly disagreed.

### 3.5. Owner Knowledge of Hygiene and Cleaning Protocols

The majority of owners (97.47%; 500) agreed or strongly agreed that poor sanitation and hygiene can lead to health problems in guinea pigs. The majority of owners strongly agreed or agreed that a disinfectant should be used when cleaning the hutch (72.89%; 371). The majority of owners agreed (54.27%; 235) that uneaten food could be a source of bacterial contamination, with 26.79% (116) strongly agreeing that this was the case. A high proportion of respondents (76.17%; 390) agreed or strongly agreed that owner hygiene could be a factor associated with the development of bacterial infections in guinea pigs.

### 3.6. Owner Knowledge of Bacterial Pathogens

The majority of respondents (91.73%; 388) correctly identified *Streptococcus pneumoniae* as a respiratory pathogen, (8.27%; 35) incorrectly attributed it to a different system (gastrointestinal etc.) and the rest of the respondents did not answer the question. The majority of respondents correctly identified predisposing factors (e.g., changes in temperature) to pneumonia in guinea pigs (64.68%; 271), with (35.32%; 148) incorrectly identifying the factors and the rest of the respondents not answering the question. In addition, most respondents were able to identify the clinical signs (nasal discharge, dyspnoea, etc.) of pneumonia with 90.78% (384) giving a correct response and 9.22% (39) selecting incorrect signs. The rest of the respondents elected not to answer the question. The majority of respondents (54.52%; 229) were not able to correctly identify other species of pets which may harbour a subclinical *Bordetella bronchiseptica* infection, with 45.48% (191) correctly identifying other species (e.g., rabbits) and the rest of respondents skipping the question. The majority of participants (56.87%; 236) were able to identify the clinical signs of *Bordetella bronchiseptica* infection in guinea pigs (anorexia, inappetence, nasal and ocular discharge, dyspnoea and sudden death). Of the respondents, 43.13% (179) failed to identify the clinical signs and the rest of the owners skipped the question.

The majority of respondents (86.47%; 358) were able to correctly identify individuals who might be predisposed to the development of bacterial enteritis (pregnant sows etc.), with 13.53% (56) failing to identify predisposed individuals and the rest of respondents opting not to answer this question. The majority of participants (91.69%; 375) correctly identified the mechanism of transmission (contaminated food or water) of *Salmonella* infection, with 8.31% (34) selecting an incorrect route of transmission and the remainder of respondents opting not to answer the question. The majority of participants (89.05%; 366) correctly identified the key clinical signs of *Salmonella* infection (fever, lethargy, anorexia and rough fur), with 10.95% (45) selecting incorrect clinical signs and the rest of the owners opting not to answer the question. The majority of participants (75.98%; 310) correctly identified methods of preventing bacterial enteritis in guinea pigs (disinfection of the environment etc.), with 24.02% (98) selecting incorrect methods.

### 3.7. Owner Knowledge Score

The mean knowledge score for the respondents was 16.45 ± 5.56, the minimum was −5.00 and the maximum achieved was 28.00. There was no significant effect of length of guinea pig ownership on knowledge score (X^2^_4_ = 2.914, *p* = 0.572). There was no significant effect of age on knowledge score (X^2^_5_ = 3.144, *p* = 0.678). There was no significant effect of sex on knowledge score (W = 7022, *p* = 0.314). There was no significant difference in knowledge score between those respondents that had or had not owned a guinea pig that had been clinically diagnosed with a bacterial infection (W = 31246, *p* = 0.475).

## 4. Discussion

This study found that 39.31% of respondents owned a guinea pig that had been clinically diagnosed with a bacterial infection. Conversely, a previous study of 1000 guinea pigs reported a much lower prevalence of bacterial infections identifying forty individuals (4.0%) with respiratory disease and whilst guinea pigs with gastrointestinal disease were presented (10 cases; 1.0%), the conditions were not bacterial in origin [3]. The low prevalence of bacterial infections in previous studies could be explained by owners practicing good husbandry by not keeping their animals in dusty or damp conditions, or indoors during the colder months [24]. Alternatively, owners may be disregarding or misinterpreting clinical signs of bacterial infections and thus not presenting their guinea pig to the veterinary practice [11,25]. Bacterial infections are sometimes subclinical, therefore the number of guinea pigs affected may be even higher than reported in this study [13]. However, we did not clinically examine the guinea pigs or perform susceptibility testing of samples, as in previous studies [3,26]. Therefore, the data collected may be influenced by owners misreporting the diagnoses provided by their veterinarian and as such should be interpreted with caution. The number of surveys completed by pet owners in this study was not representative of the guinea pig population as a whole and therefore further research, perhaps utilising veterinary records, is required. In this study, we did not question owners on the type of treatment their guinea pig had received, therefore given the limited number of licensed pharmaceuticals and risk of dysbiosis associated with the use of some antimicrobials in guinea pigs [15], it would be interesting to assess the type and efficacy of treatment prescribed. 

There was no significant effect of any demographic factors on whether the respondent had owned a guinea pig that had been reportedly clinically diagnosed with a bacterial infection. There was no effect of where guinea pigs were obtained from which was surprising, as it could be speculated that guinea pigs obtained from a rescue might be more subject to disease due to poor husbandry prior to adoption [11]. Length of guinea pig ownership also had no significant effect, it could be suggested that more experienced owners would be more likely to utilise preventative measures to mitigate the development of disease but conversely having owned guinea pigs for longer they would be more likely to have experienced a range of pathologies in their pets. In this study, we did not investigate whether there was an effect of the number of guinea pigs housed together on the development of bacterial infections. It would be expected that larger groups might be more likely to transmit infections; however, housing guinea pigs alone may elicit stress in a social species thus predisposing disease development. Group size and housing would be interesting variables to investigate in future work.

### 4.1. Upper Respiratory Tract Infections

The most commonly reported infection in this study was respiratory, with URTIs forming 46.95% of reported infections. Just over half of the owners surveyed (54.52%) were not aware of other species that can harbour *Bordetella bronchiseptica* subclinically, so guinea pigs may contract this pathogen from close contact with other species such as rabbits [4,11]. In a survey of 102 rabbits it was identified that five rabbits that were kept with guinea pigs as companions [21]. Whilst this was a low percentage of the total number of rabbits surveyed, six rabbits in the sample population were found to have nasal discharge when clinically examined despite their owner never having reported experiencing this condition. If owners cannot detect subtle signs of respiratory disease in rabbits, pathogens can be transmitted in multi-species households. Interestingly, most owners did not feel that extra insulation was necessary for guinea pigs living outdoors. Whilst the need for additional protection may depend on the cage type and the weather, fluctuations in temperature and draughts have been implicated in the development of respiratory diseases in guinea pigs [4]. Stress can also result in recrudescence of clinical signs of respiratory disease in sub-clinical carrier animals, therefore, avoiding stressful husbandry situations is of utmost importance [4].

Lower respiratory infections were less commonly reported in the sample population forming 4.69% of infections reported, however, when combined with URTIs, respiratory infections formed over half over the total number of infections reported (51.64%). Guinea pig adenovirus can cause respiratory disease [27], and therefore some of the cases reported may have been viral or involved secondary opportunistic bacterial invasion.

### 4.2. Gastrointestinal Infections

Gastrointestinal infections were reported in 11.73% of guinea pigs in the sample population. Contaminated food has been suggested as a factor associated with the frequency of gastrointestinal infections, and it is advised that owners wash fresh fruits and vegetables prior to feeding [28,29], but bacterial transmission could also occur through shared food in multi-animal environments. However, the majority of owners surveyed demonstrated a high level of awareness of good hygiene and husbandry. In this study, the majority of respondents strongly agreed or agreed that disinfectant was necessary when cleaning cages. Whilst studies have examined industry grade disinfectants and antiseptics for use in small animal hospitals [30,31], to the authors’ knowledge, no study has investigated the efficacy of commercial small animal cleaning products for use in a domestic environment. It would be of interest as to whether the aforementioned pathogens are susceptible to common pet disinfectants. A further cause of gastrointestinal problems in guinea pigs can be sudden changes to diet [29]; we did not question owners on whether they had changed their pet’s diet in this survey, but this is something that could be explored in future work.

### 4.3. Urinary Tract Infections

Guinea pigs that had reportedly been diagnosed with UTIs represented 47.83% (33) of the “other” category, and 15.49% (33) of all infections reported. A category for UTI was not initially created on this questionnaire due to concerns that confusion with other related issues such as urolithiasis might lead to inaccurate responses. Furthermore, cystitis has not been identified as a common problem in guinea pigs in previous literature, with urological disorders in general (including uroliths) accounting for only 4.20% of conditions observed in one study [3]. Whilst urinary disorders have been reported by some authors to be common in rodents, obstructions with calculi are a recognised problem in guinea pigs with bacterial infections occurring secondarily [32]. Although owners may have misidentified the problem their pet had suffered from, the high percentage of owners reporting urinary tract infections suggests that this issue requires further investigation. A limitation of using owner questionnaires is that whilst owners were explicitly asked to report clinical diagnoses from their veterinarian, owners still may have misconstrued what a bacterial infection is. Whilst retrospective studies of veterinary records allow for an accurate clinical diagnosis, the infrequent presentation of exotic pets at veterinary practices can result in a small sample size compared to similar studies of cats and dogs. The survey approach used in this study permitted a larger sample size and information on owner knowledge and husbandry practices which is not usually available from veterinary practice data.

### 4.4. Owner Knowledge

Owners demonstrated knowledge and understanding of key aspects of health and husbandry which is consistent with recent research [22]. There was no significant effect of any of the recorded demographic factors on the overall knowledge score nor was there a significant difference in knowledge between owners that had or had not owned a guinea pig that had been clinically diagnosed with a bacterial infection. It could be hypothesised that owner knowledge has a minimal impact on the prevalence of bacterial infections seen in pet guinea pigs; however, this questionnaire was targeted at guinea pig forums, possibly reaching a more knowledgeable subset of owners. Research in rabbits has captured knowledge of prospective owners at point of sale as a means of mitigating this issue [20]. The majority of participants in this study had owned guinea pigs for more than four years suggesting that they were experienced owners and likely knowledgeable of correct husbandry practices.

## 5. Conclusions

The number of owner-reported bacterial infections were higher than expected in this study. Owners appeared to be well informed on key aspects of guinea pig health and husbandry, suggesting poor husbandry may not be the only contributing factor to the development of bacterial infections. Further research is warranted, including clinical examination and microbial culture, to appreciate the impact of bacterial infections on the health and welfare of pet guinea pigs.

## Figures and Tables

**Table 1:** Statements on health and husbandry and responses ^1^.

Statement	Correct/Incorrect	Strongly Disagree % (*n*)	Disagree % (*n*)	Neither Agree nor Disagree % (*n*)	Agree % (*n*)	Strongly Agree % (*n*)	Total Answered Question (*n*)	Missing (*n*)
A guinea pig should live in a well-ventilated area	Correct	3.91 (20)	0.98 (5)	4.89 (25)	35.03 (179)	55.19 (282)	511	13
Wire mesh is the most appropriate flooring for a hutch or run	Incorrect	80.59 (411)	11.37 (58)	5.88 (30)	0.98 (5)	1.18 (6)	510	14
It is acceptable for guinea pigs to come into contact with other species of rodent	Incorrect	45.81 (235)	35.87 (184)	13.06 (67)	4.29 (22)	0.97 (5)	513	11
It is acceptable to keep guinea pigs and rabbits together if they get on	Incorrect	61.33 (314)	23.44 (120)	8.59 (44)	5.66 (29)	0.98 (5)	512	12
Guinea pigs should have companions providing dominance is not being expressed	Correct	1.17 (6)	4.30 (22)	16.60 (85)	37.50 (192)	40.43 (207)	512	12
Forage and vegetables do not have to be served at room temperature	Incorrect	8.59 (44)	28.13 (144)	33.79 (173)	26.76 (137)	2.73 (14)	512	12
Moving guinea pigs between environments (e.g., inside and outside on a weekly basis) does not cause stress induced diseases	Incorrect	0 (0)	36.59 (187)	31.31 (160)	10.57 (54)	21.53 (110)	511	13
Washing fruit and vegetables prior to feeding is not important	Incorrect	40.00 (204)	29.61 (151)	15.10 (77)	10.98 (56)	4.31 (22)	510	14
Guinea pigs can be housed outside without requiring extra insulation	Incorrect	1.37 (7)	1.37 (7)	2.73 (14)	24.22 (124)	70.31 (360)	512	12
Poor hygiene and sanitation are factors that can lead to poor health in guinea pigs	Correct	1.36 (7)	0.19 (1)	0.97 (5)	19.69 (101)	77.78 (399)	513	11
Bacterial infections are a concern for guinea pig health	Correct	1.36 (7)	1.56 (8)	7.60 (39)	34.70 (178)	54.78 (281)	513	11
A disinfectant must be used when cleaning out the hutch/cage	Correct	2.36 (12)	9.04 (46)	15.72 (80)	45.19 (230)	27.70 (141)	509	15
Uneaten food can be a source of bacterial infection	Correct	0.69 (3)	3.00 (13)	15.24 (66)	54.27 (235)	26.79 (116)	433	91
Owner hygiene can be a factor associated with bacterial infections in guinea pigs	Correct	0.97 (5)	2.86 (15)	19.92 (102)	51.76 (265)	24.41 (125)	512	12

^1^ Incorrect and correct statements presented to respondents in the questionnaire, and the percentage (%) and number of people (*n*) who rated their agreement.
